# Skin- and Plasmaautofluorescence in hemodialysis with glucose-free or glucose-containing dialysate

**DOI:** 10.1186/s12882-016-0418-0

**Published:** 2017-01-05

**Authors:** Bernd Ramsauer, Gerwin Erik Engels, Reindert Graaff, Aleksandar Sikole, Stefan Arsov, Bernd Stegmayr

**Affiliations:** 1 Department of Public Health and Clinical Medicine, Umea University, Umea, Sweden; 2 HaemoScan BV, Groningen, The Netherlands; 3 UMC Staff and Department of Endocrinology, University Medical Center Groningen and University of Groningen, Groningen, The Netherlands; 4 University Ss Cyril and Methodius, Hospital of Nephrology, Skopje, Republic of Macedonia; 5 University Ss Cyril and Methodius, Institute for Epidemiology and Biostatistics, Skopje, Republic of Macedonia; 6 Department of Nephrology, Skaraborgs Hospital, 541 58 Skövde, Sweden

**Keywords:** Haemodialysis, Plasma autofluorescence, Skin autofluorescence, Glucose free dialysate

## Abstract

**Background:**

Haemodialysis (HD) patients suffer from an increased risk of cardiovascular disease (CVD). Skin autofluorescence (SAF) is a strong marker for CVD. SAF indirectly measures tissue advanced glycation end products (AGE) being cumulative metabolites of oxidative stress and cytokine-driven inflammatory reactions. The dialysates often contain glucose.

**Methods:**

Autofluorescence of skin and plasma (PAF) were measured in patients on HD during standard treatment (ST) with a glucose-containing dialysate (*n* = 24). After that the patients were switched to a glucose-free dialysate (GFD) for a 2-week period. New measurements were performed on PAF and SAF after 1 week (M1) and 2 weeks (M2) using GFD. Nonparametric paired statistical analyses were performed between each two periods.

**Results:**

SAF after HD increased non-significantly by 1.2% while when a GFD was used during HD at M1, a decrease of SAF by 5.2% (*p* = 0.002) was found. One week later (M2) the reduction of 1.6% after the HD was not significant (*p* = 0.33). PAF was significantly reduced during all HD sessions. Free and protein-bound PAF decreased similarly whether glucose containing or GFD was used. The HD resulted in a reduction of the total PAF of approximately 15%, the free compound of 20% and the protein bound of 10%. The protein bound part of PAF corresponded to approximately 56% of the total reduction. The protein bound concentrations after each HD showed the lowest value after 2 weeks using glucose-free dialysate (*p* < 0.05). The change in SAF could not be related to a change in PAF.

**Conclusions:**

When changing to a GFD, SAF was reduced by HD indicating that such measure may hamper the accumulation and progression of deposits of AGEs to protein in tissue, and thereby also the development of CVD. Glucose-free dialysate needs further attention. Protein binding seems firm but not irreversible.

**Trial registration:**

ISRCTN registry: ISRCTN13837553. Registered 16/11/2016 (retrospectively registered).

## Background

Cardiovascular morbidity and mortality are increased in patients with decreasing kidney function [1, 2] and end-stage renal disease (ESRD). HD patients have a five-fold shorter life expectancy than age-related healthy persons [3, 4]. One factor to consider is glucose that binds to amino residues forming glycated Schiff bases, with later rearrangements forming a more stable but still reversible Amadori product. Over time, these products undergo rearrangements including crosslinking to become irreversible advanced glycation end products (AGEs). Thereby both circulating and tissue proteins, as well as lipids and nucleic acids, may be glycated and crosslinked with collagen in the skin and other tissues [5, 6].

AGEs are considered as uremic toxins and contribute to cardiovascular complications of HD patients [7, 8]. AGEs accumulate more in HD because of increased production by oxidative stress caused by the dialysis per se [9] and lowered elimination by the impaired kidneys [10]. Skin autofluorescence (SAF) is related to the accumulation of AGE and is one of the strongest prognostic markers of mortality in these patients [8]. SAF is an indirect marker for glucose degradation products [8], present not only in the skin but also in other tissues [11].

Consumption of specific foods is associated with increased AGEs [12–14]. Another source of exposure to glucose in HD patients might be the use of glucose-containing dialysate. The use of glucose in dialysate has changed several times in the history of dialysis. In the early days of HD treatment, the use of dialysates containing a high glucose concentration was important to achieve an effective osmotic ultrafiltration [15]. Later, the use of ultrafiltration by hydrostatic pressure was developed and found superior [16]. The use of glucose in the dialysate decreased and many dialysis units switched to a glucose free concentrate [17]. However, the disadvantage of non-glucose-containing dialysate was the increased risk for hypoglycemia in patients with insulin dependent diabetes mellitus and lack of a valuable addition to energy in malnourished HD patients [14, 18–21].

In previous studies, we were able to show that a single session of HD significantly reduced plasma autofluorescence (PAF) but not SAF [22]. The use of either high-flux versus low-flux dialyzers did not change SAF after HD as well [23]. Apparently, changes in plasma fluorescence did not influence SAF, marker of accumulated tissue AGEs.

Furthermore, our previous studies were performed using glucose containing dialysates. This raised the question if dialysate glucose per se could increase the load of AGE in the body.

Therefore, the aim of this study was to investigate whether PAF and SAF, reflecting the current and accumulated amount of AGEs, respectively, were influenced by the use of either glucose free or glucose-containing dialysate.

## Methods

### Study design

A longitudinal interventional study was performed at the hemodialysis center at the University Hospital in Umeå.

### Demography

During the observation period, 24 patients on chronic HD were included in the study (17 male/ 7 female). The median age was 70.5 years (range 42–85). The median vintage of HD was 52.5 months (range 11–121 months). The main reasons for HD were primary glomerular disorders (*n* = 5), diabetes nephropathy (*n* = 5, 2 with diabetes mellitus type 1, and 3 with type 2), polycystic kidney disease (*n* = 2), hypertension and/or renovascular disease (*n* = 5), postrenal cause (*n* = 2) and other or unknown diagnoses (*n* = 5). The comorbidity of the patients included hypertension (*n* = 20, 83%) and cardiovascular disease (*n* = 9, 38%). Five of these patients had suffered from myocardial infarction (21%) and 1 patient from stroke (4%). Lifestyle factors included current or previous tobacco use (15/23, 63% of whom 13% current users; missing information in 1) and alcohol consumption (wine and beer in 38%). All patients were on chronic HD with the median treatment duration/session of 4 h (range 3–5.5 h). Two patients had dialysis with low flux dialyzers (FX10), all others received dialysis with high flux dialyzers (FX80, Fresenius Medical Care, Bad Homburg, Germany).

The study was performed as a longitudinal study. Patients were informed consecutively, and those who accepted to participate were included. Exclusion criteria were an ongoing infection and inability to understand information. Patients with diabetes mellitus prone to hypoglycemia would have been excluded if they would have considered participating in the study. No such patient was present. All patients were on chronic HD during standard treatment using a dialysate with a final glucose concentration of 5 mmol/L (Biosol A201.25 glucose 5 and Biosol A301.25 glucose 5, Meda AB, Solna, Sweden). After that, the patients were switched to a glucose-free dialysate (SK-F 209, K^+^ 2 mmol/l, Ca^++^ 1,5 mmol/l, Mg^++^ 0,5 mmol/l, no glucose, Fresenius, Bad Homburg, Germany) for 2 weeks (six sessions). The dialyses within the frame of the study were performed at the same time and same weekday each time (morning dialyses).

Measurements were performed on plasma autofluorescence (PAF) and skin autofluorescence (SAF) both before and after standard HD (ST) at the end of March or the first week of April. The glucose-free dialysate period that began at the first week in May with measurements after 1w of HD (M1) and 2w of HD (M2) with GFD.

Skin-AF was obtained as a median of three measurements along the same forearm at slightly different positions. Measurements were done in a semi-dark environment at room temperature on each occasion. Each patient was his/her own control with the same conditions throughout the study except the dialysate.

### AGE reader

The AGE Reader (DiagnOptics Technologies BV, Groningen, The Netherlands) illuminates a skin surface of ~ 4 cm^2^, guarded against surrounding light, with a light source that mainly provides UV-A light between 350 and 420 nm (peak wavelength 370 nm). Autofluorescence and reflected light from the skin were measured simultaneously using a spectrometer within the instrument. Skin AF was measured in arbitrary units (AU). The arbitrary unit is based on the ratio of the average light intensity per nm in the range between 420 and 600 nm, and the average light intensity per nm in the range between 300 and 420 nm. Version 2.3 of the AGE Reader software was used. The AGE Reader had been validated and was more extensively described in previous studies [24, 25]. A good correlation between the skin AF and the tissue levels of pentosidine, *N*_ε_ –carboxy-methyl-lysine (CML) and *N*_ε_-carboxy-ethyl-lysine (CEL) was found in DM patients and age-matched healthy controls [26].

### Plasma autofluorescence (PAF)

Plasma samples were collected before and after HD for the analysis of PAF and albumin concentration. The samples were kept at −70 °C until analysis. Plasma AGEs were quantified using fluorescence with an excitation wavelength of 370 nm and an emission wavelength of 465 nm on a Tecan Genios microplate reader (Tecan Group Ltd., Männedorf, Switzerland). Total PAF was measured according to a modified protocol of Schwedler et al. [27]. In brief, plasma samples were diluted 50 times in phosphate buffered saline before measuring fluorescence as described above. The non-protein-bound fluorescence or free plasma fluorescence was determined according to a modified protocol of Wrobel et al. [28]. Thereby, plasma samples were diluted 25 times in 0.15 M trichloroacetic acid, precipitating proteins. After eliminating the precipitate by centrifugation, the fluorescence of the supernatant was measured as above. The bound plasma fraction was calculated as the difference between total and free PAF.

When fluid is removed from the patient by ultrafiltration, cells and larger molecules such as albumin are not dialyzed out of the plasma, leading to hemoconcentration. For adjusting such effects on PAF, The ratio of change in serum albumin after versus before HD was used [29, 30] to adjust PAF after dialysis for such effects.

In this study, we did not investigate other effects of glucose-free dialysis such as potassium removal or levels of triglycerides. Individual dialysates were prescribed and prepared by each dialysis device. No central container for dialysis fluid was used. Bacterial growth and endotoxin content of dialysis water supply are regularly controlled according to European regulations.

### Statistical analysis

Paired statistical analyses were performed with the Wilcoxon nonparametric test between the 3 sample periods. A two-tailed p-value of less than 0.05 was considered significant. Mean values and standard error of the mean (±) are given. Univariate correlation analyses were performed using the Spearman test (rho) to adjust for the effect of eventual outliers. SPSS statistical software (version 20 SPSS, Inc. Chicago, IL) was used for the analyses.

## Results

### Plasma autofluorescence (PAF)

The various concentrations of PAF are given in Table 1.

**Table 1:** Plasma autofluorescence mean values and standard deviation (SD, kUnit/ml) in total plasma and given as free- and protein bound

	N	Total PAF	SD	Free PAF	SD	Protein-bound PAF	SD
ST Start	24	1841	±298	584	±170	1258	±226
ST End	24	1586	±219	460	±884	1126	±163
M1 Start	24	1858	±263	558	±109	1300	±205
M1 End	24	1644 ^a)^	±182	454	±49	1189 ^a)^	±158
M2 Start	21	1738 ^b)^	±236	551	±82	1187 ^a,b)^	±222
M2 End	21	1518 ^c)^	±170	463	±34	1056 ^a,b)^	±162
ST End adj	24	1519	±262	440	±94	1078	±196
M1 End adj	24	1633 ^a)^	±253	453	±79	1180	±193
M2 End adj	21	1410 ^c)^	±205	430	±52	980	±173
Δ ST	24	−255	±170	−124	±119	−131	±130
Δ M1	24	−214	±132	−103	±72	−111	±119
Δ M2	21	−219	±170	−89	±54	−131	±164
Δ ST adj	24	−322	±221	−144	±133	−178	±151
Δ M1 adj	24	−225	±175	−105	±85	−120	±138
Δ M2 adj	21	−328	±244	−121	±69	−206	±212
Δ % ST	24	−13.2	±7.8	−18.7	±12.4	−9.5	±9.4
Δ % M1	24	−11.0	±6.1	−17.1	±8.6	−7.8	±9.3
Δ % M2	21	−12.0	±8.6	−15.1	±7.5	−9.7	±13.5
Δ % ST adj	24	−15.9	±10.9	−21.9	±15.3	−13.6	±10.8
Δ % M1 agj	24	−11.9	±8.4	−17.8	±11.7	−8.9	±10.0
Δ % M2 adj	21	−18.1	±12.7	−21.1	±10.3	−15.8	±16.5

Δ = the difference in PAF as the subtraction of PAF at the End – Start value; adj = the value is adjusted to the change in plasma concentration by i.e. fluid intake or removal by dialysis. A ratio is achieved between the plasma albumin concentration at the end versus at the start of HD. The PAF at end is corrected by dividing with this value; ^a)^ *p* < 0.05 compared to March, ^b)^ *p* < 0.05 compared to May 1, ^c)^ *p* < 0.01 compared to May 1

There was a significant reduction of all three plasma compounds (Total, free and protein bound PAF) after each dialysis (*p* < 0.001). Comparing the reduction (Δ PAF after - Δ PAF before dialysis) of the total, free-, protein bound PAF there was no difference in reduction by dialysis at the first measurement (ST) versus M1 (period with glucose free dialysate) or 1 week later (M2).

For total PAF there was no difference between the predialysis values at ST versus M1 and M2 while the M2 values were lower than M1 (*p* = 0.019). The total- PAF values after HD, adjusted for the effect of ultrafiltration showed a higher value at M1 than at ST (*p* = 0.015) but a lower value than at M2 (*p* = 0.001). The value after HD at M2 was not significantly lower than the value at ST (*p* = 0.058).

For the protein bound PAF there was no difference between the pre-HD concentration at ST versus M1. The concentration at M2 was lower than at ST (*p* = 0.027). The M2 concentration was also lower than the M1 (*p* = 0.005). The protein bound non-adjusted and adjusted PAF values after each HD showed the lowest value after 2 weeks using glucose-free dialysate (*p* < 0.05). The findings can be interpreted that a reduction in protein-bound PAF occurs during the glucose-free dialysate period.

For the free PAF concentration, there was no difference between the ST, M1, and M2 neither the pre-HD values, the differences (before versus after HD) nor the post HD values.

Table 2 shows that the free PAF compound represents approximately 30% of the total PAF both before and after dialysis and the protein bound correspondingly about 70%. The dialysis resulted in a reduction of the total PAF of approximately 15%, the free compound of 20% and the protein bound of 10%. Since the protein bound part was more than twice as large as the free PAF, the reduction of the protein bound part of PAF represented at a median 56% of the overall reduction.

**Table 2:** Distribution of mean values (in %) of free and bound PAF fractions

	Free part of PAF	Protein-bound part of PAF	Δ % free PAF under dialysis	Δ % protein bound PAF under dialysis	Fraction of the plasma-bound part of reduction
ST Start	31.7%	68.3%			
ST End	29.0%	71.0%	−18.7%	−9.5%	0.51
M1 Start	30.0%	70.0%			
M1 End	27.6%	72.4%	−17.1%	−7.8%	0.52
M1 Start	31.7%	68.3%			
M2 End	30.5%	69.5%	−15.1%	−9.7%	0.60
ST END adj	28.9%	71.1%	−21.9%	−13.6%	0.55
M1 End adj	27.7%	72.3%	−17.8%	−8.9%	0.53
M2 End adj	30.5%	69.5%	−21.1%	−15.8%	0.63
mean	29.7%	70.3%	−18.6%	−10.8%	0.56

Δ % = the percentage decrease in p-AF as the division of p-AF at the End /Start value

The SAF pre-dialysis values (ST, M1, M2) were not related to the pre-HD values of PAF neither to the total (rho = 0.395, *p* = 0.056), free (rho = 0.317, *p* = 0.13) or to the protein bound PAF (rho = 0.22, *p* = 0.30) nor to the change in PAF that appeared after HD (Total: rho = −0.26, *p* = 0.27; free: rho = −0.25, *p* = 0.24; rho = −0.07, *p* = 0.75).

There was a strong correlation between total, protein-bound and free -PAF values before and after HD and also at different sampling times, such that high values were maintained high and vice versa (rho ≥0.57, *p* ≤ 0.007). The changes in total, protein-bound and free PAF after dialysis were related to the start value (rho ≥ −0.60, *p* < 0.005) such that a high initial value resulted in a greater reduction after HD.

There were correlations between total PAF and protein bound PAF (rho >0.74, *p* < 0.01) and total versus free PAF during the ST and M1 series (rho >0.57, *p* < 0.005) but not M2 series. No correlation was found between protein-bound PAF and free PAF.

There was no correlation of PAF at the start of the study and age (rho = 0.016, *p* = 0.938).

### Skin autofluorescence (SAF)

SAF was measured before and after HD for the various time points and the difference of SAF before and after dialysis (Table 3).

**Table 3:** Skin autofluorescence mean values and standard deviation (SD, arbitrary units AU)

	N	SAF start	SD	SAF end	SD	Δ end - start	Δ %
ST	24	3.949	±0.615	3.985	±0,654	0.036	−1,2
M1	24	4.173	±0.626	3.957	±0.647	−0.216	5.2
M2	24	4.177	±0.651	4,096	±0.695	0.081	1.6

When the pre-dialysis SAF values at ST, M1 and M2 were compared, there was a significant increase in SAF from the first measurement (ST) to the investigation 1 month later (M2) by a median of 4.8% (*p* = 0.032). However, when comparing the value achieved after the standard HD (with glucose-containing dialysate) with the pre-dialysis SAF value at M1 (after the first period with glucose free dialysate), there was no significant difference between the values.

There was a nonsignificant (*p* = 0.61) increase of 1.2% in SAF after the HD with glucose-containing dialysate (ST) comparing with SAF before the same HD. At M1, using glucose-free dialysate, the SAF after HD was reduced by 5.2% (*p* = 0.002). One week later (M2) the reduction of 1.6% after HD was not significant (*p* = 0.33). There was no significant difference between the values comparing the reduction (Δ SAF end - SAF start) at M1 versus M2 on glucose free dialysis.

For all three series (ST, M1, M2) there was a strong correlation between SAF values before and after HD and also at different sampling times such as that high values were maintained high and vice versa (rho > 0.68, *p* < 0.001). The changes in SAF after a dialysis session were not related to the initial values. Skin AF measured at the start of the study (ST) did not correlate with age (rho = 0.255, *p* = 0.23).

The length of the hemodialysis sessions (at a mean 4.3 h, ±0.6, range 3–5.5) did not correlate with the change in SAF nor the change in any PAF.

Blood glucose was monitored if symptoms appeared. 2 patients, both with diabetes type 2 and insulin treatment developed a hypoglycemia. One of the patients got a bolus injection of glucose, the other was treated with oral dextrose. No patient received or required glucose infusion in parallel to dialysis.

## Discussion

In the present investigation of hemodialysis with glucose free hemodialysis (GFD) there appeared a significant decrease, after dialysis, of SAF and total and protein bound PAF concentrations. PAF and SAF reflect the current and accumulated amount of AGEs. SAF is an indirect risk factor for CVD [8, 11], this indicates that the load of protein bound AGEs seems to decrease with a prolonged treatment period with a GFD. Notably, the free part of PAF was not influenced by the GFD. Thus, the difference in outcome in protein bound PAF using GFD versus glucose-containing dialysate may well be due to a fast transfer of the glucose from the dialysate into the blood and extravascular space where a degradation and conversion into glucose degradation products may occur. Initial less tight attachment to various molecules including proteins may result in reversible Amadori products.

The presence of less tight bonds is indicated by the reduction of protein bound PAF in plasma but also by the decrease of SAF after dialysis after glucose-free HD in the present study. The main glucose degradation products are pentosidine and *N*_ε_ –carboxy-methyl-lysine (CML), that in vitro are water soluble and dialyzable, of a molecular weight of less than 400D. However, both AGEs are considered as protein-bound uremic toxins [31, 32] and poorly removed by HD [33], as confirmed by the present study. Another reason explaining the poor reduction of the free PAF could be an increased production of GDP molecules throughout the dialysis due to the oxidative stress induced by glucose [34]. The more pronounced reduction of SAF by HD at M1 than M2 may indicate that there were more reversibly bound glucose degradation products in the tissue at M1, for example Amadori products. One week later the clearance of such products was less effective, indicating the presence of a lower ratio of reversibly bound AGEs.

The reduction of protein bound PAF levels after 2 weeks of glucose-free dialysis favor the concept of a local extravascular formation of AGEs.

The lack of relation between SAF and PAF, found in this study, is in congruence with others [27, 35].

In contrast to GFD, the use of glucose-containing HD did not change SAF after dialysis, either during the present study or previous studies despite the use of high flux dialyzers [22, 23]. It is discussed that a higher glucose concentration in the dialysate may impair inflammation [17], lipid levels [36], hyperglycemia and conversion of carbohydrates into glucose degradation products and AGEs that subsequently lead to oxidative stress [37].

Hypoglycemia may develop with glucose free dialysate. This risk is most crucial for patients with insulin dependent diabetes mellitus prone to hypoglycemia. Therefore glucose free dialysis seem less suitable for such patients. The use of a glucose infusion in parallel to dialysis may be preventive if the patient does not compensate hypoglycemia by eating.

## Conclusions

The present study shows that a glucose free dialysate may result in a significant reduction of SAF, as a marker of AGEs and Amadori products, in contrast to when using glucose-containing dialysate. The protein bound parts of PAF also showed a decrease after 2 weeks. This indicates that it may be possible to hamper or even reverse the deposits of AGEs in tissue. Future longitudinal studies with glucose free dialysate can help to clarify if this leads to a reduction of SAF and in limits the progress of CVD in HD patients.

## Abbreviations

AGE: Advanced glycation end products
CVD: Cardiovascular disease
ESRD: End-stage renal disease
GFD: Glucose free dialysate
HD: Haemodialysis
M1: Measurement after 1 week with glucose-containing dialysate
M2: Measurement after 2 weeks with glucose-containing dialysate
PAF: Plasma autofluorescence
SAF: Skin autofluorescence
ST: Time of first measurement standard treatment with glucose-containing dialysate
